# Transport of Organic Contaminants in Composite Vertical Cut-Off Wall with Defective HDPE Geomembrane

**DOI:** 10.3390/polym15143031

**Published:** 2023-07-13

**Authors:** Hai Lin, Wenzhou Huang, Liangni Wang, Zhanlei Liu

**Affiliations:** 1 School of Infrastructure Engineering, Nanchang University, Nanchang 330031, China; linhai@ncu.edu.cn (H.L.); 406000210033@email.ncu.edu.cn (W.H.); 2 Key Laboratory of Ministry of Education for Geomechanics and Embankment Engineering, Hohai University, Nanjing 210024, China; liu_zhanlei@hhu.edu.cn

**Keywords:** vertical cut-off wall, defect of geomembrane, organic contaminants, the migration behavior of contaminants, analytical solution

## Abstract

Soil-bentonite vertical cut-off wall is an emergency technique used for contaminant control in geo-environmental engineering, high-density polyethylene (HDPE) geomembrane (GM) with an extremely low-permeability coefficient is expected to enhance the contaminant barrier effect of the vertical cut-off wall. To evaluate the barrier performance of the composite barrier composed of GM and soil-bentonite mixture towards organic contaminant, while also quantitively revealing the impact of GM defects and placement, a one-dimensional transport model for organic contaminants in composite barrier is solved under semi-infinite boundary conditions. The proposed transport model is validated by numerical simulations using COMSOL Multiphysics 5.4, and the effects of GM defect rate, placement within the composite isolation wall, and contact level with soil-bentonite on contaminant transport behavior are further studied. The results show that as the average frequency of GM defects increases from 2.5 to 50 holes per hectare, the breakthrough time of organic contaminants through composite barrier decreases by almost 70%. Poor contact level between GM and soil-bentonite mixture may reduce the breakthrough time of the composite cut-off wall by 65%. Although the selection of GM placement has limited impact on the transient flux of contaminants, it does affect the total flux of contaminants over a certain period of time. The effects of permeability coefficient, effective diffusion coefficient, distribution coefficient, and hydraulic head of the composite cut-off wall can be considered by the proposed analytical solution, which would provide guidance and reference for the design and service performance evaluation of the composite cut-off wall.

## 1. Introduction

The employment of a liner barrier constitutes a crucial engineering intervention aimed at curtailing the spread of organic contaminants to the surrounding ecosystem, particularly in regions including but not limited to landfill sites, mining smelter ponds, and industrial parks [1]. Overall, there are two types of liner barrier systems: horizontal impermeable liners widely used in landfills, e.g., composite liners consisting of high-density polyethylene geomembrane (GM), geosynthetic clay liner (GCL), clay, and low-permeability vertical impermeable barriers, e.g., grouting curtains or composite barriers fabricated of soil, bentonite, and cement [2]. Vertical impermeable cut-off wall is an emergency measure that can effectively slow down the horizontal transport of contaminants, and the permeability coefficient, diffusion coefficient, and adsorption characteristics of contaminants in liner materials will affect the transport process [3]. High-density polyethylene (HDPE) GM has an extremely low-permeability coefficient, and its application in vertical impermeable barriers is expected to further enhance the contaminant barrier effect of the liner [4].

The investigation of contaminant transport and the barrier effects of liners is a topic of great importance to engineers and researchers. The diffusion coefficient of organic contaminants in liner materials plays a critical role in determining breakthrough time, and the utilization of low-permeability GM + GCL composite liner can be challenging due to its inherent difficulty in taking full advantage of its low-permeability coefficient [5]. Wu et al. (2016), Pu et al. (2017), and Xie et al. (2013, 2015) [6,7,8,9] derived the concentration variation law of organic contaminants in three-layer composite liners (i.e., GM + GCL + Soil Liner) and the corresponding solutions were obtained based on assumptions such as zero flux and semi-infinite boundaries. According to these theoretical solutions or numerical calculations, sensitivity analysis of various parameters for controlling contaminant transport can be carried out successfully [10]. These research studies on vertical transport behavior of contaminates in horizontal impermeable liners can provide references for calculating contaminant transport in the vertical impermeable cut-off wall [11]. Neville et al. (2006) [12] established a steady-state transport model of contaminants in vertical impermeable cut-off wall and compared it with numerical simulation results. Li et al. (2017) [13] provided a one-dimensional transient analytical solution for contaminant transport in a single-layer vertical impermeable cut-off wall and provided a design chart for nondimensionalized effluent contaminant flux by analyzing the study. Acar et al. (1990), Peng et al. (2020), and Xie et al. (2020) developed transient diffusion analytical models. Studies have shown that the selection of boundary conditions has a significant impact on transport calculations, and reasonable selection of boundary conditions is a prerequisite for transport calculations [14,15,16].

The defect of GM cannot be ignored, and the impact of leakage on contaminant transport should be clarified. Lee et al. (2000) [17] found through experiments that the permeability coefficient of GM in composite liner can reach $1\times 10^{-5}\;m/s$ when the contact between the GM and the surrounding medium is imperfect. Barroso et al. (2006) [18] studied the contact problem of vertical impermeable cut-off wall and surrounding media in three-scale tests and found that convection in impermeable barriers with GM cannot be ignored in contaminant transport. Philip et al. (2001) [19] found that under low hydraulic heads as well as convection is one of the main factors causing contaminant transport through vertical impermeable cut-off wall. Zhang et al. (2010) [20] considered convection, diffusion, and adsorption effects and studied the influence of the thickness, permeability coefficient, and design depth of vertical impermeable curtains on contaminant transport. They found that convection is the dominant factor for contaminant transport and dispersion under high hydraulic heads. From existing research, it can be inferred that the combined use of thin GM and soil-bentonite vertical impermeable cut-off wall can enhance the contaminant barrier effect of the liner.

In this study, considering convection, diffusion, and adsorption effects, a one-dimensional transport model of organic contaminants in a composite vertical impermeable cut-off wall with defective GM and soil-bentonite barrier was established. The mathematical physical equation method was used to obtain the analytical solution of the model, and then the calculation results of the analytical solution were compared with the calculation results of the numerical simulation method to verify the accuracy of the model. Then, further analysis was conducted on the influence of parameters such as GM defects, location, and adsorption capacity on contaminant transport in the vertical impermeable cut-off wall.

## 2. Calculation Model

### 2.1. Geometric Model

**Figure 1:**
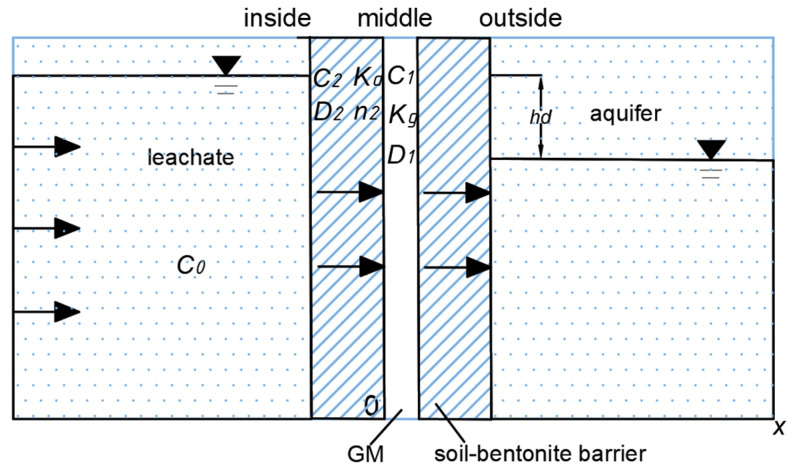
Contaminant transport model diagram.

The schematical diagram of the organic contaminant transport in composite vertical cut-off wall with defective GM is shown in Figure 1. As shown in Figure 1, the vertical composite impermeable cut-off wall comprises a soil-bentonite barrier and a geomembrane. In consideration of the convective, diffusive, and adsorptive effects of contaminants by the impermeable barrier, both convection and diffusion phenomena transpire in the two layers, and adsorption of the contaminant occurs in the soil-bentonite barrier.

### 2.2. Basic Assumptions

To obtain an analytical solution for the one-dimensional migration of organic contaminants in the vertical contaminant barrier, the model adopts the following assumptions based on previous theoretical foundations and related experimental studies: (1) the contaminant in the filtration liquid is a single organic contaminant and undergoes one-dimensional migration in the horizontal direction; (2) the vertical impermeable cut-off wall remains undeformed with a constant pore volume, and the geomembrane contains holes; (3) the contaminant migration considers convection, diffusion, and adsorption effects, and contaminant concentration in the leachate is assumed to be constant at *C*_0_; (4) molecular diffusion follows Fick’s second law, and the contaminant adsorption is an isothermal linear process that has reached equilibrium; (5) the barrier and aquifer are homogeneous and isotropic, and under the head of the filtration liquid, the seepage reaches a steady state.

### 2.3. Governing Equations and Auxiliary Conditions

Considering the convection and diffusion effects of contaminants, the flux of contaminants along the x direction can be expressed as:

(1) $$f=nvC-nD\frac{\partial C}{\partial x}$$

where *f* is the contaminant flux, *n* is the total porosity of soil, *v* is the seepage velocity, *C* is the contaminant concentration, and *D* is the molecular diffusion coefficient.

In the geomembrane, contaminants mainly undergo convection and diffusion. Considering its relatively thin thickness, here it is assumed to be in a steady-state transport. The steady-state transport governing equation for the GM is:

(2) $$D_{1}\frac{\partial^{2}C_{1}(x)}{\partial x^{2}}-v_{1}\frac{\partial C_{1}(x)}{\partial x}=0$$

where *C*_1_ is the contaminant concentration at any position *x* within the geomembrane, *D*_1_ is the effective diffusion coefficient of the GM, and *v*_1_ is the average linear velocity of the contaminant in the geomembrane.

The soil-bentonite barrier has a low permeability, which can prevent the penetration of contaminants and adsorb contaminants within a limited thickness. According to previous assumptions and the law of conservation of mass, the transient one-dimensional transport governing equation of contaminants in the soil-bentonite barrier can be expressed as follows.

(3) $$R_{d}\frac{\partial C_{2}(x,t)}{\partial t}=D_{2}\frac{\partial^{2}C_{2}(x,t)}{\partial x^{2}}-v_{2}\frac{\partial C_{2}(x,t)}{\partial x}$$

where *R_d_* is the retardation factor of the soil-bentonite barrier [21], *C_2_* is the contaminant concentration at any position *x* in the soil-bentonite barrier at any time t, *D_2_* is the effective diffusion coefficient of the soil-bentonite barrier, and *v_2_* is the average linear velocity of the contaminant in the soil-bentonite barrier.

(4) $$R_{d}=1+\frac{\rho_{d}K_{d}}{n_{2}}$$

where *ρ_d_* is the dry density of the soil-bentonite barrier; *n*_2_ is the porosity of the soil-bentonite barrier; and *K_d_* is the distribution coefficient of soil-bentonite barrier.

Assuming that the background concentration of contaminants in the vertical cut-off wall is zero and the redistribution process of contaminants at the interface between the infiltration liquid and the GM is instantaneous, the boundary at the interface between leachate and GM can be expressed as follows:

(5) $$C_{1}(0)=K_{g}C_{0}$$

(6) $$C_{2}(x,0)=0\;(x>0)$$

The continuity conditions between the GM and the soil-bentonite barrier are:

(7) $$C_{1}(x){|}_{x=L_{1}}=K_{g}^{\prime }C_{2}(x,t){|}_{x=L_{1}}$$

(8) $$D_{1}\frac{\partial C_{1}(x)}{\partial x}{|}_{x=L_{1}}=n_{2}D_{2}\frac{\partial C_{2}(x,t)}{\partial x}{|}_{x=L_{1}}$$

where *K_g_* is the concentration distribution coefficient between the GM and the adjacent medium, and *K’_g_* is equal to *K_d_* [22].

When considering convection in contaminant transport, boundary conditions have little effect on the transport results [23]. Here, we assume that the outer boundary of the soil-bentonite barrier is a semi-infinite boundary:

(9) $$\frac{\partial C_{2}(\infty ,t)}{\partial x}=0$$

## 3. Model Solving and Verification

### 3.1. Analytical Solution

The general solution of the governing Equation (2) is:

(10) $$C_{1}(x)=k_{1}e^{r_{1}x}+k_{2}$$

where *k*_1_ and *k*_2_ are undetermined parameters, here we set *r*_1_= *v*_1_*/D*_1_ and *r*_2_= *v*_2_*/D*_2_. By substituting the Equation (5) and the Equation (7) into Equation (10):

(11) $$k_{1}=\frac{K_{g}^{\prime }C_{2}(L_{1},t)-K_{g}C_{0}}{e^{r_{1}x}-1}$$

(12) $$k_{2}=\frac{K_{g}C_{0}e^{r_{1}x}-K_{g}^{\prime }C_{2}(L_{1},t)}{e^{r_{1}x}-1}$$

Substituting Equations (10)–(12) into the Equation (8):

(13) $$C_{2}(L_{1},t)=C_{0}+m\frac{\partial C_{2}(L_{1},t)}{\partial x}$$

(14) $$m=\frac{e^{r_{1}L_{1}}-1}{K_{g}r_{2}e^{r_{1}L_{1}}}$$

For the governing Equation (3), we use Laplace transform method to solve it:

(15) $$U(x,p)=\int_{0}^{\infty }C_{2}(x,t)e^{-pt}dt$$

where *U*(*x*, *p*) is the Laplace transform of *C*_2_(*x, t*) with respect to *t* and *p* is the transform parameter.

Taking Laplace transform on both sides of the governing Equation (3):

(16) $$R_{d}pU(x,p)=D_{2}\frac{\partial^{2}U(x,p)}{\partial^{2}x}-v_{2}\frac{\partial U(x,p)}{\partial x}$$

whose general solution is:

(17) $$U(x,p)=k_{3}e^{\lambda_{1}x}+k_{4}e^{\lambda_{2}x}$$

where *k*_3_ and *k*_4_ are constants, and expressions of *λ*_1_ and *λ*_2_ are as follows:

(18) $$\lambda_{1}=\frac{r_{2}}{2}+\sqrt{\frac{r_{2}^{2}}{4}+\frac{pR_{d}}{D_{2}}}$$

(19) $$\lambda_{2}=\frac{r_{2}}{2}-\sqrt{\frac{r_{2}^{2}}{4}+\frac{pR_{d}}{D_{2}}}$$

Laplace transform of the Equations (9) and (13):

(20) $$\frac{\partial U(\infty ,p)}{\partial x}=0$$

(21) $$U(L_{1},p)=\frac{C_{0}}{p}+m\frac{\partial U(L_{1},p)}{\partial x}$$

By substituting Equations (20) and (21) into Equation (17):

(22) $$k_{3}=0$$

(23) $$k_{4}=\frac{C_{0}}{pe^{\lambda_{2}L_{1}}(1-m\lambda_{2})}$$

Substituting the obtained parameters into Equation (17):

(24) $$U(x,p)=\frac{C_{0}e^{\left[(L_{1}-x)(\sqrt{\frac{r_{2}^{2}}{4}+\frac{pR_{d}}{D_{2}}}-\frac{r_{2}}{2})\right]}}{p[1+m(\sqrt{\frac{r_{2}^{2}}{4}+\frac{pR_{d}}{D_{2}}}-\frac{r_{2}}{2})]}$$

Applying the inverse Laplace transform principle and related properties to Equation (24), we can obtain:

(25) $$\begin{array}{l} \frac{2C_{2}(x,t)}{C_{0}}=erfc\left(\frac{x-\frac{v_{2}t}{R_{d}}}{2\sqrt{\frac{D_{2}t}{R_{d}}}}\right)+e^{r_{1}L_{1}+r_{2}x}erfc\left(\frac{x+\frac{v_{2}t}{R_{d}}}{2\sqrt{\frac{D_{2}t}{R_{d}}}}\right)-\\ e^{\frac{r_{2}x}{4}(e^{r_{1}L_{1}}+\frac{v_{2}t}{R_{d}x}-1){\mathrm{csch}}^{2}\left(\frac{r_{1}L_{1}}{2}\right)}(e^{r_{1}L_{1}}+1)erfc\left[\frac{x+\frac{v_{2}t}{R_{d}}\coth\left(\frac{r_{1}L_{1}}{2}\right)}{2\sqrt{\frac{D_{2}t}{R_{d}}}}\right] \end{array}$$

Substituting Equation (25) into Equation (1) yields the expression of contaminant flux:

(26) $$\frac{2f}{n_{2}v_{2}C_{0}}=erfc\left(\frac{x-\frac{v_{2}t}{R_{d}}}{2\sqrt{\frac{D_{2}t}{R_{d}}}}\right)-e^{\frac{r_{2}x}{4}(e^{r_{1}L_{1}}+\frac{v_{2}t}{R_{d}x}-1){\mathrm{csch}}^{2}\left(\frac{r_{1}L_{1}}{2}\right)}\coth\left(\frac{r_{1}L_{1}}{2}\right)erfc\left[\frac{x+\frac{v_{2}t}{R_{d}}\coth\left(\frac{r_{1}L_{1}}{2}\right)}{2\sqrt{\frac{D_{2}t}{R_{d}}}}\right]$$

where all the undetermined coefficients and parameters have been determined. This is the process of solving the one-dimensional transport governing equation of organic contaminants.

### 3.2. Comparison with Numerical Method

The application of numerical methods in engineering is quite common. In order to further verify the accuracy of the analytical model, this paper uses the numerical simulation software COMSOL for simulation research, and the related calculation parameters are listed in Table 1. The obtained calculation results are compared and verified with the analytical solution in this paper.

**Figure 2:**
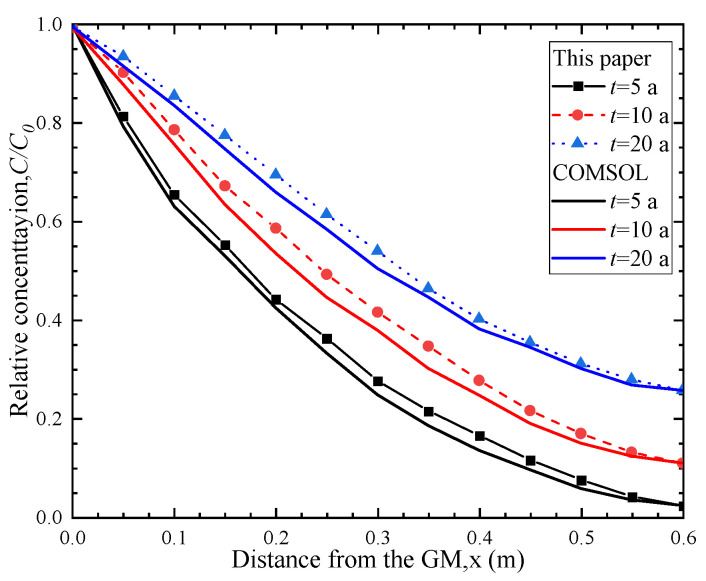
Comparison of the proposed analytical model in this paper and the numerical method.

Figure 2 shows the comparison between the analytical model in this paper and the numerical results. It can be seen from the Figure 2 that when *t* = 5 a, 10 a and 20 a, the calculation results of the analytical model in this paper are in good agreement with the concentration distribution curve obtained by the numerical simulation software. This further validates the correctness of the analytical model proposed in this paper.

## 4. Analysis of Parameter

Based on the one-dimensional transport model established in this article, the impacts of convection, diffusion, and adsorption on the transport of organic contaminants in the composite cut-off wall were systematically studied. The effects of model parameters on the transport of organic contaminants in this vertical cut-off wall were comprehensively analyzed. Benzene, which has a strong migration ability, was selected as the single organic contaminant in landfill leachate [24]. Assuming that the hydraulic head difference *h_d_* on both sides of the vertical barrier is 0.3m and the initial concentration of benzene *C*_0_ is 1.63 mg/L, the breakthrough criterion was set to be 0.1, which means that the barrier was considered to be breached when the ratio of the outside concentration of the vertical cut-off wall to the initial concentration reached 0.1.

Considering the situation where GM has holes, the simplified formula for calculating the volume leakage rate of the vertical barrier is provided by the following equation [25].

(27) $$Q=\frac{2h_{d}L_{w}}{l}[kb_{w}+\sqrt{kl\theta }]$$

where *h_d_* is the hydraulic head difference, *L_w_* is the connected fold lengths, *l* is the thickness of adjacent media, *k* is the permeability coefficient of adjacent media, *b_w_* is the half-width of the folds, *θ* is the transmissivity between GM and adjacent media. The expressions for excellent contact, good contact, and poor contact conditions are provided by Equations (28) to (30), respectively [26].

(28) $$\log_{10}\theta =-0.321+1.036(\log_{10}k)+0.0180{\left(\log_{10}k\right)}^{2}$$

(29) $$\log_{10}\theta =0.07+1.036(\log_{10}k)+0.0180{\left(\log_{10}k\right)}^{2}$$

(30) $$\log_{10}\theta =1.15+1.092(\log_{10}k)+0.0207{\left(\log_{10}k\right)}^{2}$$

Assuming that the hole frequency *m_a_* on the GM is expressed per hectare, the Darcy flux through the barrier is provided by the following equation [27].

(31) $$v_{a}=\frac{m_{a}Q}{A}$$

where *A* is the cross-sectional area of the area under investigation.

According to the law of mass conservation, the relationship between the seepage velocity in the GM and the soil-bentonite barrier is provided by the following equation:

(32) $$v_{a}=v_{1}=n_{2}v_{2}$$

In this study, the fold length *L_w_* = 500 m. The thickness *l* and permeability coefficient *k* of the soil-bentonite barrier were used. The half-width of the folds is *b_w_* = 0.1 m, and the transmissivity is *θ =* 3.2 × 10^−9^ m^2^ [28]. Other parameters were referenced from the literature [29,30], and the corresponding calculation parameters are shown in Table 1.

### 4.1. Influence of Holes

**Figure 3:**
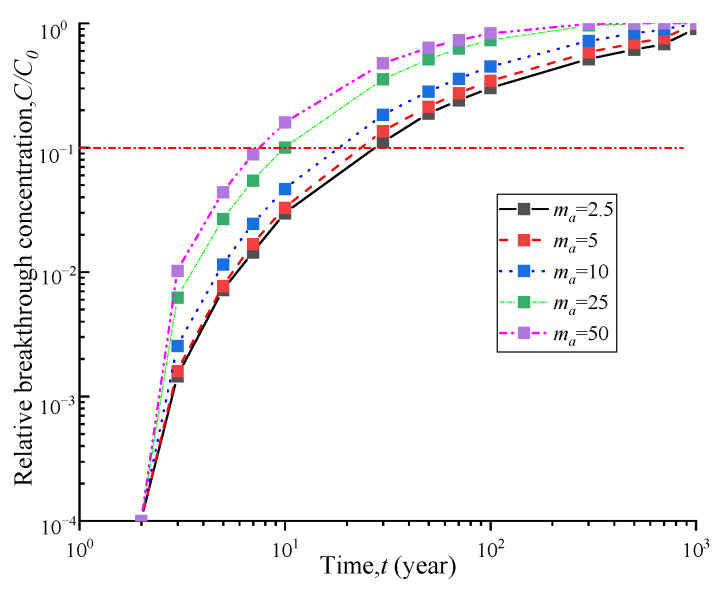
Breakthrough curves of different hole frequencies.

**Figure 4:**
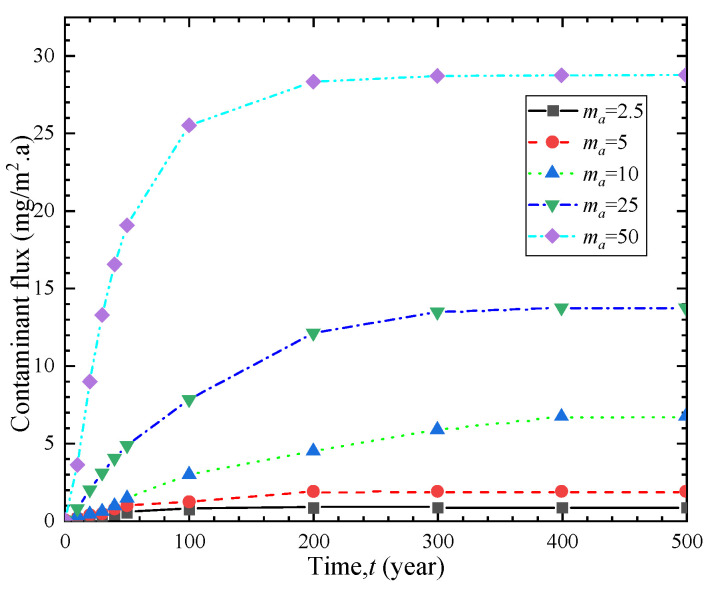
Contaminant flux curves of different hole frequencies.

**Figure 5:**
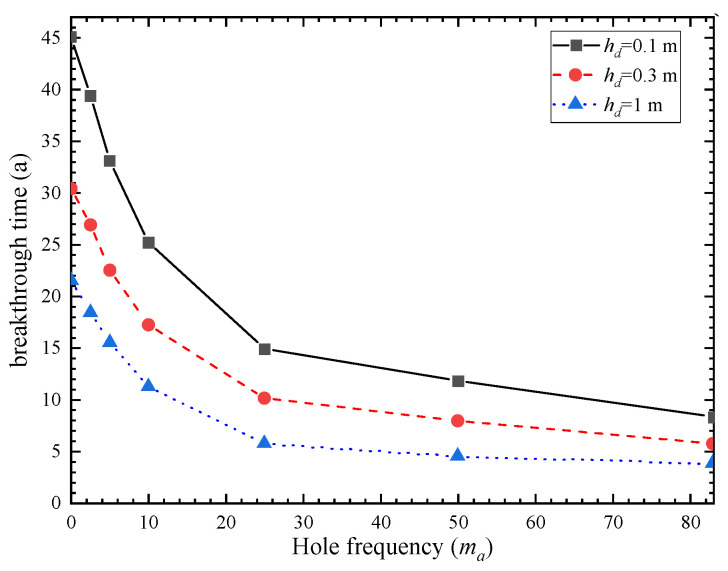
Breakthrough time curves of different hole frequencies.

The phenomenon of geosynthetic liner with holes is quite common and can be caused by various factors. The shape and size of the holes are also diverse. The hole frequencies on GM were 4 and 22 holes/ha respectively under good and poor construction quality with an average hole area of approximately 1 cm^2^ [31]. Figure 3 and Figure 4 demonstrate the breakthrough curve and contaminant flux curve of vertical cut-off wall under different hole frequencies. The breakthrough time of the vertical cut-off wall was obtained by considering hole numbers of 2.5, 5, 10, 25, and 50 holes per hectare of GM, which were 26.9 a, 22.5 a, 17.2 a, 10.1 a, and 7.9 a, respectively. Increasing the hole frequency from 2.5 to 5, 10, 25, and 50 resulted in a reduction in breakthrough time by 16%, 36%, 62%, and 70%, respectively. Figure 4 shows that as the hole frequency increases, the contaminant flux also significantly increases. Figure 5 shows the breakthrough time curves under different hole frequencies and three hydraulic head conditions. It can be observed that the breakthrough time of the composite barrier decreases as the hole frequency and hydraulic head increase. These results indicate the critical significance of controlling the number of holes on GM for the contaminant prevention performance of vertical cut-off wall.

### 4.2. Influence of Interface Transmissivity

**Figure 6:**
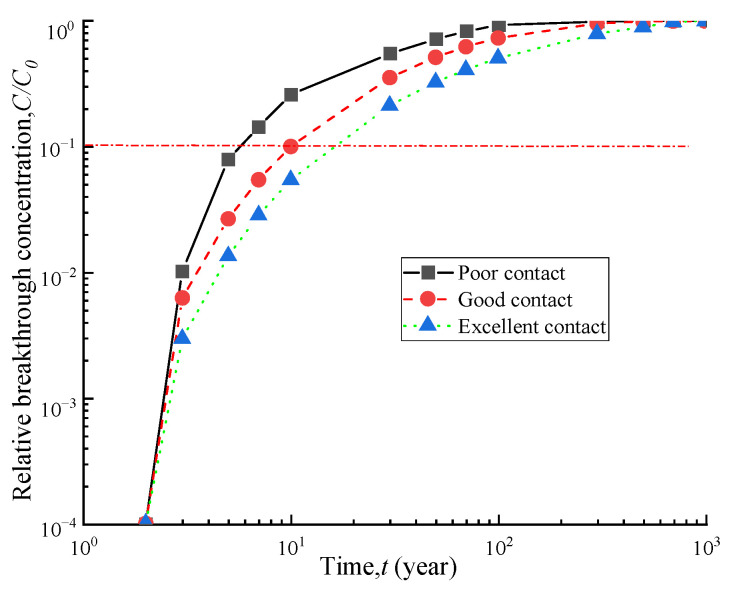
Breakthrough curves of different contacts.

The interface hydraulic conductivity between the GM and adjacent media can affect the amount of leakage, and its magnitude is determined by the contact conditions. Figure 6 shows the breakthrough curves of a GM with a soil-bentonite barrier under three different contact conditions. The breakthrough times of the vertical barrier were 5.8 a, 10.1 a, and 16.6 a for the three different contact conditions. Compared with the excellent contact condition, the breakthrough times decreased by 39% and 65% for the good and poor contact conditions, respectively. Therefore, controlling the contact conditions reasonably during construction is essential to improve the anti-leakage performance of contaminant prevention barriers.

### 4.3. Influence of GM Location

**Figure 7:**
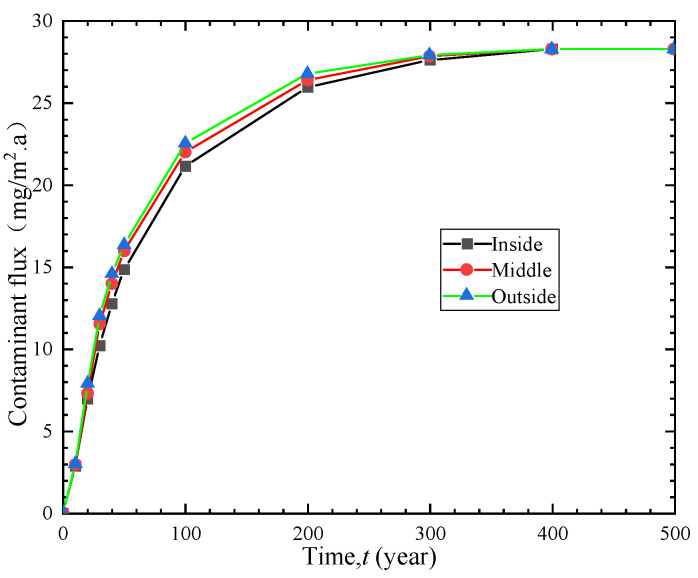
Effect of GM location on contaminant flux.

In order to investigate the influence of GM location on the anti-seepage performance of vertical cut-off wall, the organic contaminant benzene was selected as the target contaminant, and the GM was studied and analyzed under three different placement conditions. Figure 7 shows the temporal variations in contaminant flux under the inside placement condition (GM near the inlet boundary of the contaminant), middle placement condition, and outside placement condition (GM near the outlet boundary of the contaminant). The contaminant flux curves under the three conditions are quite similar and eventually tend to be equal, but the total contaminant flux for each condition varies within a certain time period. Under the internal placement condition, the total contaminant flux is minimized, while it is maximized under the external placement condition. Therefore, selecting internal placement is more reasonable under these conditions. Since choosing the GM location during construction does not increase the construction difficulty or engineering cost, making a reasonable choice of the GM location during construction is of great significance for improving the anti-seepage performance of vertical cut-off wall.

### 4.4. Influence of Soil-Bentonite Barrier Thickness

**Figure 8:**
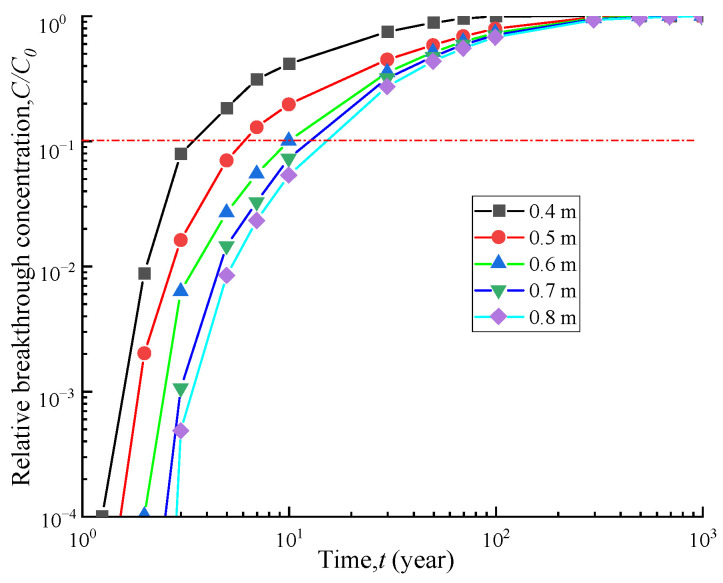
Breakthrough curves of different cut-off wall thicknesses.

**Figure 9:**
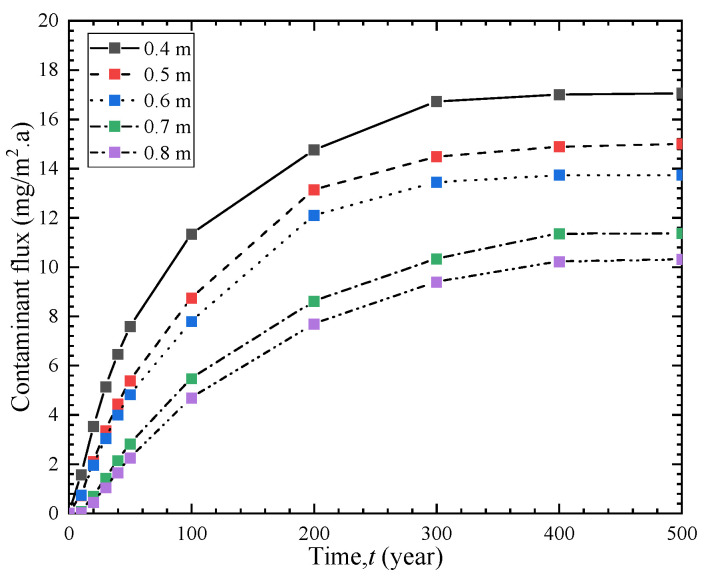
Contaminant flux curves of different cut-off wall thicknesses.

Figure 8 illustrates the effect of the thickness of the soil-bentonite barrier on the breakthrough time of the entire vertical cut-off wall. For vertical cut-off wall thicknesses of 0.4 m, 0.5 m, 0.6 m, 0.7 m, and 0.8 m, the corresponding breakthrough times are 3.8 a, 6.1 a, 10.1 a, 12.9 a, and 15.5 a, respectively. As the barrier thickness increases from 0.4 m to 0.5 m, 0.6 m, 0.7 m, and 0.8 m, the breakthrough time increases by 60%, 165%, 239%, and 307%, respectively. Moreover, Figure 9 shows that as the barrier thickness increases, the effluent contaminant flux at the outlet boundary decreases. This indicates that a thicker barrier has a stronger blocking effect, leading to better contaminant prevention performance. However, the engineering cost also increases accordingly. Therefore, during construction, considerations must be made regarding both economic costs and anti-seepage performance to achieve the optimal solution.

### 4.5. Influence of Leachate Heads

**Figure 10:**
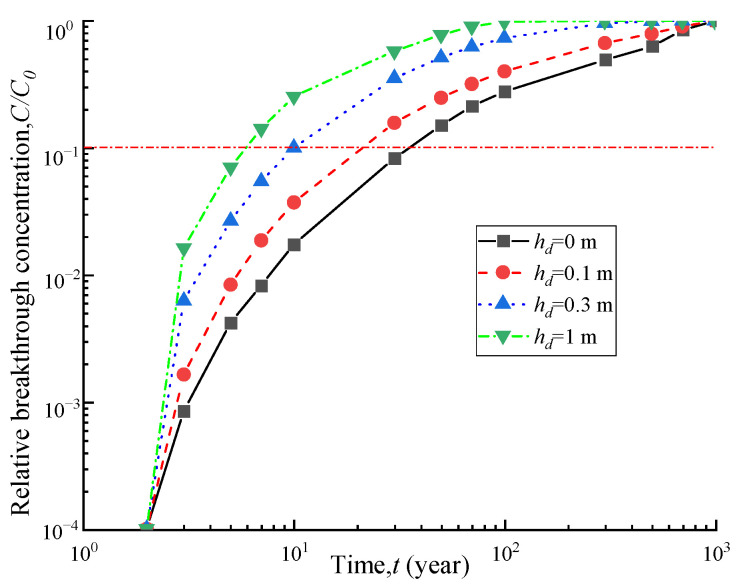
Breakthrough curves under different leachate heads.

**Figure 11:**
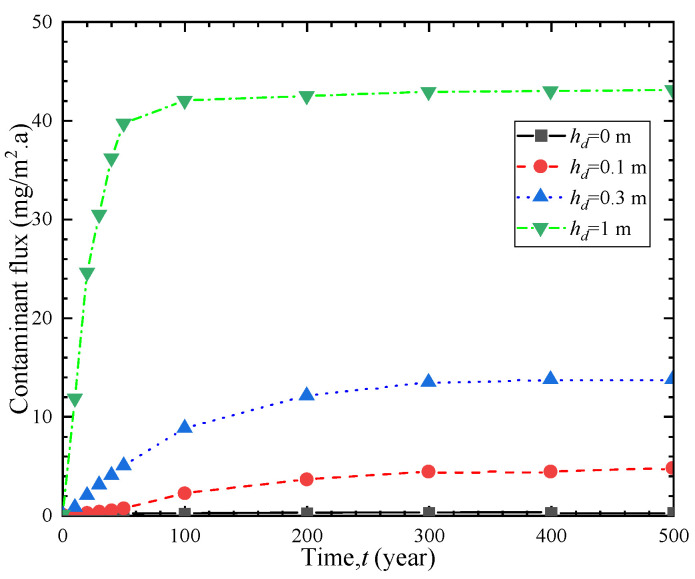
Contaminant flux curves under different leachate heads.

To investigate the effect of percolation fluid head on the one-dimensional transport of contaminants, Figure 10 and Figure 11 depict the breakthrough curve and effluent flux curves of contaminants under different leachate heads. As shown in Figure 10, when the percolation leachate head is 0 m, 0.1 m, 0.3 m, and 1 m, the corresponding breakthrough times are 35.1 a, 22.0 a, 10.1 a, and 5.7 a, respectively. When the percolation leachate head increases from 0 m to 0.1 m, 0.3 m, and 1 m, the breakthrough time decreases by 37%, 71%, and 83%, respectively. Figure 11 shows that as the percolation leachate head increases, the effluent contaminant flux at the outlet boundary significantly increases. These findings indicate that an increase in percolation leachate head accelerates the one-dimensional transport of organic contaminants, resulting in a significant increase in the outlet boundary flux. Therefore, controlling percolation fluid effectively can extend the service life of barriers in practical engineering applications.

## 5. Conclusions

Based on the semi-infinite boundary condition assumption, one-dimensional transport model of organic contaminants in composite vertical cut-off wall with defective high-density polyethylene geomembrane is established and solved numerically. The influences of GM pore ratio, GM placement position, and the contact level between GM and soil-bentonite cut-off wall on the migration law of pollutants are analyzed by the relative pollutant concentration, breakthrough time and contaminant flux. The following conclusions were mainly drawn:

(1) Compared with the defect-free geomembrane, the service life of composite barriers with the defective geomembrane is significantly reduced. When the hole frequency increases from 5 to 25 and 50, the service life decreases by 55% and 65%, respectively.

(2) The results show that the quality of the liner–soil contact significantly affects the flux of contaminants in the vertical cut-off wall. Specifically, compared with excellent contact conditions, the breakthrough time is respectively shortened by 39% and 65% under good and poor contact conditions, respectively. This influence on the transport behavior of contaminants cannot be ignored, and it is recommended that the construction process carefully control the contact conditions to ensure good performance.

(3) The choice of the geomembrane position has limited impact on the transient flux of contaminants, but it does affect the total flux of pollutants over a certain period of time. For example, when benzene is the target contaminant, the total contaminant flux is the smallest when the GM is inserted inside the soil, and it is the largest when the liner is outside the soil.

(4) Increasing the thickness of the cut-off wall can effectively extend its service life and improve its anti-contaminant effect. When the barrier thickness is increased from 0.4 m to 0.6 m and 0.8 m, the breakthrough time increases by 165% and 307%, respectively.

(5) An increase in the hydraulic head of the leachate can accelerate the one-dimensional transport process of organic contaminants and significantly increase the outflow boundary flux. When the hydraulic head of the leachate is increased from 0 m to 0.1 m, 0.3 m, and 1 m, the breakthrough time is shortened by 37%, 71%, and 83%, respectively. Therefore, it is necessary to control the hydraulic head difference of the leachate in actual engineering to ensure the service life of the composite barrier.

## Figures and Tables

**Table 1:** Calculation parameters.

Parameter	GM	Soil-Bentonite Barrier
Thickness, *L* (m)	0.0015	0.6
Porosity, *n*	*/*	0.5
Effective diffusion coefficient, *D* (m^2^/s)	3.5 × 10^−13^	9.23 × 10^−10^
Partition coefficient, *K_g_*	30	*/*
Distribution coefficient, *K_d_* (mL/g)	*/*	1
Hydraulic conductivity, *k* (m/s)	1 × 10^−12^	1 × 10^−10^
retardation factor *R_d_*	*/*	2.4

## Data Availability

Not applicable.
